# Demographics and Outcomes of Pulmonary Hypertension Patients in United States Emergency Departments

**DOI:** 10.5811/westjem.2020.2.45187

**Published:** 2020-04-16

**Authors:** Susan R. Wilcox, M. Kamal Faridi, Carlos A. Camargo

**Affiliations:** Massachusetts General Hospital, Department of Emergency Medicine, Boston, Massachusetts

## Abstract

**Introduction:**

Pulmonary hypertension (PH) is a common, yet under-diagnosed, contributor to morbidity and mortality. Our objective was to characterize the prevalence of PH among adult patients presenting to United States (US) emergency departments (ED) and to identify demographic patterns and outcomes of PH patients in the ED.

**Methods:**

We analyzed the Nationwide Emergency Department Sample (NEDS) database, with a focus on ED patients aged 18 years and older, with any International Classification of Diseases, Clinical Modification (ICD)-9-CM or ICD-10-CM diagnosis code for PH from 2011 to 2015. The primary outcome was inpatient, all-cause mortality. The secondary outcomes were hospital admission rates and hospital length of stay (LOS).

**Results:**

From 2011 to 2015, in a sample of 121,503,743 ED visits, representing a weighted estimate of 545,500,486 US ED visits, patients with a diagnosis of PH accounted for 0.78% (95% confidence interval [CI], 0.75–0.80%) of all US ED visits. Of the PH visits, 86.9% were admitted to the hospital, compared to 16.3% for all other ED visits (P <0.001). Likewise, hospital LOS and hospital-based mortality were higher in the PH group than for other ED patients (e.g., inpatient mortality 4.5% vs 2.6%, P < 0.001) with an adjusted odds ratio (aOR) of 1.34 (95% CI, 1.31–1.37). Age had the strongest association with mortality, with an aOR of 10.6 for PH patients over 80 years (95% CI, 10.06–11.22), compared to a reference of ages 18 to 30 years.

**Conclusion:**

In this nationally representative sample, presentations by patients with PH were relatively common, accounting for nearly 0.8% of US ED visits. Patients with PH were significantly more likely to be admitted to the hospital than all other patients, had longer hospital LOS, and increased risk of inpatient mortality.

## INTRODUCTION

Pulmonary hypertension (PH) is defined as pressure elevation in the pulmonary circulation with a mean pulmonary artery pressure over 25 millimeters of mercury (mmHg)^1^ and can arise from a multitude of physiologic insults resulting in increased pulmonary vascular resistance. This sustained elevation in pressure leads to strain on the right ventricle^2^ and eventual heart failure if untreated.^3^ In addition to resulting in chronic issues, PH impacts the approach to resuscitation, as common interventions such as volume administration or intubation can be deleterious in the setting of right heart failure.^2^,^4^

Despite having substantial clinical impact, PH remains under-diagnosed.^5^ Over the last 30 years, clinicians outside the emergency department (ED) have increasingly recognized the risks of PH and right ventricular failure,^2^,^6^ but this diagnosis has been underappreciated during emergency care.^4^,^7^,^8^ Quantifying the burden of PH in the ED is difficult, as it is a heterogeneous condition, with five groups defined by the World Health Organization based upon the underlying etiology.^9^ Data are sparse for the rates of patients with PH presenting to EDs, and there are no studies of the diagnosis or management of PH in the ED. The only demographic study of all groups of PH in the ED was a single-center study, finding a 0.84% prevalence of PH in ED visits.^10^ A large epidemiologic study of ED visits focused on Group 1 PH, or pulmonary arterial hypertension (PAH), a rare disease with estimates of 5–15 cases per one million adults.^11^ Yet even this rare condition was responsible for approximately 0.01% of all ED visits.^12^ The remaining literature on the assessment of PH in the ED is limited to case reports^13^,^14^ and a small observational study.^15^

Improving the care of patients with PH in the ED begins with appropriate recognition of the condition. While it may seem evident that patients with PH have higher-acuity ED presentations as compared to other patients, the magnitude of this discrepancy is unknown. Quantifying the prevalence and acuity of patients with of PH in the ED is therefore integral to designing future studies of the emergency management of PH.

Our objective was to characterize the prevalence of PH among adult patients presenting to the ED, identify demographic patterns of these patients, and to evaluate admission rates, hospital length of stay (LOS), and inpatient mortality for these patients.

## METHODS

We analyzed the Nationwide Emergency Department Sample (NEDS) database, developed for the Healthcare Cost and Utilization Project (HCUP) sponsored by the Agency for Healthcare Research and Quality and constructed annually using records from state ED databases and state inpatient databases, to collect data on all ED visits, regardless of disposition. NEDS is the largest ED database in the US, yielding national estimates of hospital-based ED visits, and providing a snapshot of demographics for selected conditions. Unweighted, it contains data from approximately 30 million ED visits each year. Weighted, it estimates roughly 135 million ED visits per year.^16^ This study was declared exempt from review by the institutional review board of Massachusetts General Hospital.

Patients included for analysis were those ages 18 years and older, with any ED visit, with a diagnosis that met the 9^th^ or 10^th^ revision of the *International Classification of Diseases, Clinical Modification* (ICD-9-CM or ICD-10-CM, respectively) codes for PH, including Groups 1–5 of PH in the first through 10^th^ diagnosis field.^9^ Patients were included if they had a code for PH as an ED diagnosis or hospital diagnosis. The list of included ICD-9-CM and ICD-10-CM codes are provided in the Supplemental File. In the HCUP outpatient databases, the first listed diagnosis is the condition considered to be chiefly responsible for the visit.

We collected data from NEDS from 2011–2015, including demographic characteristics of age, gender, and national quartile for median household income, as estimated by the patient’s home ZIP code. Primary insurance types were categorized as public (Medicare and Medicaid), private, self-pay, and other. ED visit data were reviewed, including diagnoses, ED disposition, and hospital disposition. We also reviewed hospital characteristics, such as geographic region (Northeast, South, Midwest, and West) as defined by the US Census Bureau, and annual ED visit volume, trauma center designation, urban or rural status, and teaching status.

**Population Health Research Capsule**

What do we already know about this issue?

Pulmonary hypertension (PH) is an under-diagnosed condition with high morbidity and mortality.

What was the research question?

We analyzed the national database of ED visits to assess the inpatient, all-cause mortality of PH patients.

What was the major finding of the study?

Patients with a diagnosis of PH accounted for 0.78% of all United States ED visits, with an adjusted odds ratio for mortality of 1.34.

How does this improve population health?

PH is relatively common among ED visits, and is associated with increased rate of inpatient mortality.

The primary outcome measure was inpatient, all-cause mortality. The secondary outcomes were hospital admission rates and hospital LOS. Of note, data on ED LOS, ED observation unit admission, and intensive care unit admission are not available in NEDS.

### Statistical Analysis

All analyses included appropriate inflation using sampling weights, and we estimated variance using all observations in the database to account for domain-level variance. ED visits by patients with PH were compared to all ED visits. We reported weighted frequencies and proportions with corresponding 95% confidence intervals (CI) for patient and hospital characteristics, and used chi-square test to test statistical significance. Hospital-based mortality was computed by dividing the number of ED and inpatient, any-cause deaths by the number of PH-related ED visits. Because no unique patient identifiers were provided with ED records, the unit of analysis for the study was an ED visit. We ran bivariate analyses to explore associations of inpatient death, total ED visits, ED disposition, and hospital LOS with PH visits.

We ran a multivariable logistic-regression model to test the relationship between PH ED visits and inpatient mortality. Our goal was to assess the outcomes attributable to PH, while controlling for confounders. We selected variables a priori for possible inclusion in the model; the final model was chosen using lowest Akaike’s information criterion with the following predictors: age, gender, patient’s primary health insurance, geographic location, trauma center status, and teaching status of the hospitals. To assess change over time in admission rates and inpatient mortality rates for pulmonary hypertension visits, we ran logistic regression models with year as a continuous variable. All analyses were conducted using SAS version 9.4 (SAS Institute, Cary, NC) software. A two-sided P-value of < 0.05 was considered statistically significant.

## RESULTS

### Characteristics of Study Subjects

From 2011 to 2015, there was a weighted estimate of 4,233,762 US ED visits, with an annual average of 846,752 visits among adults with PH, which accounted for 0.78% (95% confidence interval [CI], 0.75–0.80%) of all US ED visits for adults. Table 1 shows the weighted results. Patients with PH were significantly older than the entire ED cohort, with a higher percentage of visits for patients 61 years and older, and were comprised of more women, at 61.0% compared to 57.3% for all ED visits (P < 0.001). PH patients were more likely to have public insurance (84.4% vs 49.8%, P < 0.001) and have been seen at metropolitan teaching hospitals (53.0% vs 46.3%, P < 0.001). A PH code was the primary code for 118,351 visits, unweighted, for a weighted frequency of 525,904 visits (0.096%, 95% CI, 0.092–0.10).

### Main Results

Of the weighted 4,233,762 ED visits for patients with a diagnosis of PH, 86.9% were admitted to the hospital, compared to an admission rate of 16.3% for all other ED visits (P < 0.001) (Table 2). Likewise, hospital LOS was higher in the PH group than the remainder of the ED patients admitted at 6.2 days vs 4.8 days (P < 0.001), and the inpatient mortality was also higher for the PH group (4.5% vs 2.6%, P < 0.001). The rate of death in the ED was lower in the PH cohort compared to all other ED visits, at 0.13% vs 0.17% (P < 0.001). The admission rate was over 85% for all years studied, although there was a slight decrease in admission rates for PH visits between 2011 and 2015, with a peak in 2012 at 88.0% and 86.3% in 2015 (P < 0.001). The top 10 ICD-9 primary diagnosis codes for admitted patients were hearing loss (389), pneumonia (486), obstructive chronic bronchitis (49,121), acute kidney failure (5849), urinary tract infection (5990), atrial fibrillation (42,731), acute subendocardial myocardial infarction, (41,071), cerebral artery occlusion (43,491), other chest pain (78,659), and acute pancreatitis (5770).

**Figure 1:**
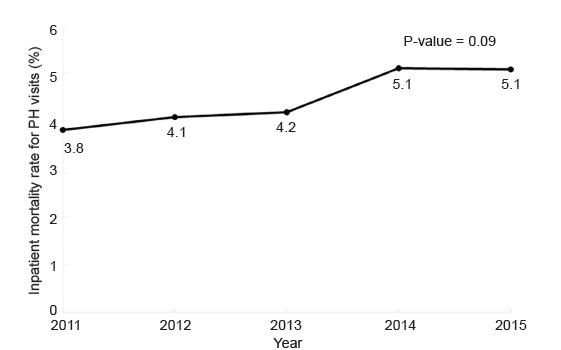
Change in inpatient mortality rate for pulmonary hypertension (PH) emergency department visits in the United States, 2011–2015. P = 0.09 for trend.

Patients with PH had an unadjusted odds ratio mortality of 1.24 (95% CI, 1.22–1.26) and an adjusted odds ratio (aOR) of inpatient mortality of 1.34 (95% CI, 1.31–1.37), compared to all other ED visits. Over the five-year period, the inpatient mortality remained relatively stable, between 3.8 and 5.1% (P = 0.09) (Figure 1). Age had the strongest association with morality, with significant increases in mortality for each decile of life, including an aOR of 10.6 for those over 80 years old (95% CI, 10.06–11.22) compared to a reference of ages 18–30 years. Visits by PH patients in metropolitan teaching hospitals and those with trauma level designation were also associated with increased mortality. Female gender and private insurance status were associated with decreased aOR for mortality (Table 3).

## DISCUSSION

In this nationally representative sample of ED visits, presentations by patients with ICD-9-CM and ICD-10-CM codes corresponding to PH were relatively common, accounting for 0.78% of weighted visits, similar to the results of a recent, single-center study.^10^ PH can arise from numerous etiologies, including idiopathic, connective-tissue disease, or drug-related causes (Group 1); left heart failure (Group 2); hypoxemic respiratory disease (Group 3); chronic thromboembolic disease (Group 4); and miscellaneous causes, such as sarcoidosis or sickle cell disease (Group 5). An older study using the NEDS database evaluated the rate of ED visits for patients with Group 1 PH,^12^ a rare condition with a reported prevalence of only 6.6–25 cases per million per year.^17^,^18^ A single-center study analyzed the demographics of all five PH groups presenting to the ED,^10^ but no study has previously assessed ED visits for patients with all five groups in a large, nationwide dataset. Present in almost 1% of all ED visits, PH is relatively common for a condition that has not been previously well described in the ED literature and is associated with significantly increased resource utilization and inpatient mortality.

In this investigation, most patients with PH were women, consistent with prior studies of PH.^10^,^12^,^19^ PH patients were significantly older than the remaining ED patient population, likely tracking with the development of PH secondary to comorbidities, such as left heart failure, hypoxic lung disease, and other chronic medical conditions. With improved treatments for PH, the life expectancy is increasing,^20^ and coupled with the aging of the population, recognizing PH in the ED will become more important. Not only does the management of PH differ from other chronic medical conditions,^4^ but among patients with comorbidities such as congestive heart failure, chronic obstructive pulmonary disease or interstitial lung disease, PH is associated with an increased attributable mortality.^21^–^29^

Patients with PH were significantly more likely to be admitted to the hospital than all other patients, at a rate of approximately 87%, similar to the previously published report of ED patients with Group 1 PH, at 82%.^12^ Likewise, other studies have found an increasing rate of hospitalizations associated with PH, including both Group 1 and secondary PH.^30^,^31^ A prior study demonstrated that the mean hospital LOS for PH increased from 5.89 days to 6.67 days (p = 0.04) between 2010 and 2013.^31^ These values are consistent with our findings for PH admissions originating from the ED, at 6.2 days, significantly longer than the average LOS for all other admissions from the ED. These findings indicate that patients with PH have high acuity in the ED. Although emergency physicians traditionally have not focused on this patient population,^4^ they recognize the acuity of their ED presentations, as they only discharge about 11%.

Accordingly, the inpatient mortality was significantly higher for patients with PH than other patients admitted via the ED, with a persistent inpatient mortality rate of 4–5% over the years studied. These findings are concordant with the previously published mortality rate for Group 1 PH patients, at 5.4%.^12^ A population-based analysis of mortality data from the National Vital Statistics System for 2001–2010 found that PH as any contributing cause of death was 5.5 per 100,000 in 2001 and 6.5 per 100,000 in 2010.^30^

Prior studies of PH have had disparate results regarding gender-related differences in mortality, with some finding increased mortality in women,^30^,^32^ and others, increased mortality in men.^12^,^33^ In the current study, while PH patients were more commonly women, men had a higher risk of inpatient mortality, consistent with prior studies based in the ED.^10^,^12^ The reason for the discrepancy in prior, gender-based findings is not clear and merits further investigation.

Not surprisingly, older age was most strongly associated with increased inpatient mortality, with significant increases in mortality for each decile of life, including an aOR of 10.6 for those over 80 years old. While intuitive, this finding has been shown in other studies of Group 1 and secondary PH alike.^30^,^34^ Visits by PH patients to metropolitan teaching hospitals and those with trauma level designation were also associated with increased mortality, likely reflecting the complexity of patients at these institutions.

Patients with PH often experience a substantial delay between the onset of symptoms and diagnosis, with one study finding a two-year lag for 21% of patients with Group 1 PH,^35^ leading to patients being diagnosed late in their course. Delay in diagnosis correlates with decreased survival.^36^ As this current study demonstrates, patients with an existing diagnosis of PH were relatively common among ED visits, and with the historical under-appreciation of PH,^5^ more undiagnosed patients may also be presenting. The ED is a major point of contact with the healthcare system for many patients,^37^ providing an opportunity for the emergency physician to consider the diagnosis and make timely referrals. The most common presenting symptom for patients with PH is dyspnea,^38^ a common and nonspecific complaint in the ED.^39^ Given this vague presentation for patients with a high-acuity condition, increased awareness among emergency physicians is essential to improving timely diagnosis.

## LIMITATIONS

The NEDS database relies on administrative rather than clinical data, and this study was not designed to reflect details of clinical care in the ED that may have affected mortality or admission rates. Second, studies based upon ICD-9-CM and ICD-10-CM codes are always at risk of classification bias, and this is a particular issue with a previously under-reported condition such as PH. A review of patients with moderate to severe PH in the VA system found that only 17% of these patients had PH documented as a diagnosis in their medical records.^5^

Other studies have shown that Group 1 PH is recorded in public records at a higher prevalence than it is at specialized centers.^19^ It is unknown whether the larger records are overestimating the prevalence or whether the specialized centers are underestimating. As PH can arise from multiple comorbidities known to be associated with increased mortality, such as left-sided heart failure and chronic obstructive pulmonary disease, these comorbidities may be responsible for the increased utilization and mortality seen in the PH cohort. However, patients with PH complicating heart failure^26^ and pulmonary disease^28^ have higher mortality that patients with those conditions without PH.

Changes in the ICD-9-CM and ICD-10-CM coding during the study period may also have affected the results. The top ICD-9 primary diagnosis codes for admitted patients refer to the indication for the outpatient visit in the HCUP database, and therefore, do not necessarily reflect the reason the patient was admitted. NEDS does not contain patient identifiers. We were therefore unable to assess the frequency of return visits, repeat admissions, or long-term outcomes.

## CONCLUSION

In this nationally representative sample of US ED visits, presentations by patients with ICD-9-CM and ICD-10-CM codes corresponding to PH were relatively common, accounting for 0.78% of visits by adults. Patients with PH were significantly more likely to be admitted to the hospital than all other patients and had an increased risk of inpatient mortality compared to all other ED visits. Older age was most strongly associated with increased inpatient mortality. With the aging of the population, recognizing PH will become increasingly important for ED clinicians. As PH often presents with only vague symptoms, emergency physicians should be aware of this common, high-acuity condition to improve timely diagnosis.

## Supplementary Information



## Figures and Tables

**Table 1:** Demographics and hospital characteristics for adult patients with pulmonary hypertension visiting United States emergency departments (ED), 2011–2015.

ED Visit and Hospital Characteristics	Sampled Unweighted ED Visits, n	PH Visits Weighted, n (%)	95% CI	All Other ED Visits Weighted, n (%)	95% CI	P-value
Overall	121,503,743	4,233,762 (0.78)	0.75–0.80	541,266,724 (99.22)	99.20–99.25	< 0.001
Age, years						< 0.001
18–30	31,633,471	67,213 (1.59)	1.49–1.69	141,853,675 (26.21)	25.96–26.46	
31–40	20,130,189	114,276 (2.70)	2.58–2.82	90,156,103 (16.66)	16.54–16.78	
41–50	19,095,656	257,371 (6.08)	5.87–6.28	85,328,858 (15.76)	15.65–15.88	
51–60	17,817,726	539,174 (12.74)	12.42–13.05	79,640,167 (14.71)	14.60–14.83	
61–70	12,909,470	793,668 (18.75)	18.53–18.97	57,234,217 (10.57)	10.48–10.67	
71–80	10,092,759	1,034,252 (24.43)	24.18–24.67	44,269,286 (8.18)	8.04–8.32	
>80	9,824,472	1,427,806 (33.72)	33.06–34.39	42,784,418 (7.90)	7.71–8.10	
Gender						< 0.001
Male	51,679,738	1,651,243 (39.00)	38.74–39.27	231,359,767 (42.74)	42.51–42.98	
Female	69,824,005	2,582,519 (61.00)	60.73–61.26	309,906,957 (57.26)	57.02–57.49	
Primary health insurance						< 0.001
Public	60,683,058	3,576,092 (84.47)	83.86–85.07	269,425,287 (49.78)	49.21–50.35	
Private	33,302,392	493,085 (11.65)	11.05–12.24	150,158,160 (27.74)	27.18–28.30	
Self-pay	20,338,141	86,003 (2.03)	1.88–2.18	89,467,529 (16.53)	15.95–17.11	
Other	7,180,152	78,582 (1.86)	1.70–2.01	32,215,748 (5.95)	5.62–6.28	
Median household income by ZIP code						< 0.001
1 (lowest)	40,894,148	1,235,970 (29.19)	27.81–30.58	180,555,833 (33.36)	32.18–34.53	
2	31,486,020	1,064,985 (25.15)	24.02–26.29	141,378,476 (26.12)	25.29–26.95	
3	26,541,369	989,032 (23.36)	22.40–24.32	119,089,347 (22.00)	21.21–22.80	
4 (highest)	19,926,113	863,663 (20.40)	18.82–21.98	88,385,486 (16.33)	15.31–17.35	
unknown	2,656,093	80,112 (1.89)	1.65–2.14	11,857,582 (2.19)	2.06–2.32	
Geographic location						0.004
Northeast	22,026,007	733,850 (17.33)	15.62–19.05	103,308,707 (19.09)	17.72–20.45	
South	24,564,967	1,027,327 (24.27)	22.04–26.49	124,582,458 (23.02)	21.55–24.49	
Midwest	52,412,565	1,756,352 (41.48)	38.91–44.06	215,506,895 (39.82)	38.00–41.63	
West	22,500,204	716,233 (16.92)	15.34–18.49	97,868,663 (18.08)	16.98–19.19	
Trauma center						0.11
No	51,678,478	1,757,596 (41.51)	39.03–44.00	232,190,969 (42.90)	41.18–44.61	
Yes	69,825,265	2,476,166 (58.49)	56.01–60.97	309,075,755 (57.10)	55.39–58.82	
Hospital teaching status						< 0.001
Metropolitan teaching	54,365,929	2,242,816 (52.97)	50.49–55.46	250,316,054 (46.25)	44.41–48.08	
Metropolitan non-teaching	47,415,858	1,532,608 (36.20)	33.87–38.53	198,375,635 (36.65)	35.05–38.25	
Nonmetropolitan	19,721,956	458,338 (10.83)	9.80–11.86	92,575,034 (17.10)	16.21–17.99	
Urban location						< 0.001
No	6,492,623	76,945 (1.82)	1.53–2.11	30,706,683 (5.67)	5.24–6.10	
Yes	115,011,120	4,156,817 (98.18)	97.89–98.47	510,560,041 (94.33)	93.90–94.76	

*CI*, confidence interval; *ED*, emergency department; *PH*, pulmonary hypertension.

**Table 2:** Outcomes for United States emergency department (ED) visits for patients with pulmonary hypertension (PH), 2011–2015.

Outcome	PH	All Other ED Visits	P-value
ED Disposition, n (weighted %)			
Discharged	98,270 (10.44)	92,972,703 (77.26)	< 0.001
Admitted to hospital	820,892 (86.89)	19778120 (16.28)	< 0.001
Death in ED	1,230 (0.13)	209,273 (0.17)	< 0.001
Hospital LOS (days), mean (95% CI)	6.21 (6.13–6.28)	4.83 (4.78–4.88)	< 0.001
Inpatient mortality, n (weighted %)	36,708 (4.51)	517,463 (2.63)	< 0.001

*CI*, confidence interval; *ED*, emergency department; *LOS*, length of hospital stay; *PH*, pulmonary hypertension

**Table 3:** Association of pulmonary hypertension (PH) emergency department (ED) visits with inpatient mortality in the United States, 2011–2015.

Variables	aOR	95% CI	P-value
PH disease			
No	1 (Reference)		
Yes	1.34	1.31–1.37	< 0.001
Age			
18–30	1 (Reference)		
31–40	1.39	1.34–1.44	< 0.001
41–50	2.39	2.29–2.50	< 0.001
51–60	3.97	3.79–4.16	< 0.001
61–70	5.69	5.41–5.97	< 0.001
71–80	7.38	7.01–7.78	< 0.001
> 80	10.63	10.06–11.22	< 0.001
Gender			
Male	1 (Reference)		
Female	0.78	0.77–0.78	< 0.001
Primary health insurance			
Public	1 (Reference)		
Private	0.90	0.88–0.92	< 0.001
Self-pay	1.02	0.98–1.05	0.36
Other	0.96	0.91–1.02	0.15
Geographic location			
Northeast	1 (Reference)		
South	0.88	0.84–0.92	< 0.001
Midwest	0.92	0.88–0.96	< 0.001
West	1.10	1.05–1.16	< 0.001
Trauma center			
No	1 (Reference)		
Yes	1.13	1.09–1.18	< 0.001
Hospital teaching status			
Metropolitan teaching	1 (Reference)		
Metropolitan non-teaching	0.85	0.82–0.89	< 0.001
Nonmetropolitan	0.89	0.85–0.93	< 0.001

*aOR*, adjusted odds ratio; *CI*, confidence interval; *PH*, pulmonary hypertension.
