# Supplementary material for: Demographics and Outcomes of Pulmonary Hypertension Patients in United States Emergency Departments
Source: West J Emerg Med. 2020 Apr 16;21(3):714–21. doi: 10.5811/westjem.2020.2.45187 (PMC7234722; doi:10.5811/westjem.2020.2.45187)
Supplement: Supplementary file 1 [file wjem-21-714-s001.docx]

**Supplemental Material:**

Inclusion International Codes for Diagnosis for Pulmonary Hypertension Cohort:

**ICD-9-CM codes:**416.0, 416.1, 416.2, 416.8, 416.9, 417.9, 745.4

**ICD-10-CM codes:**I27.0, I27.1, I27.2x, I27.81, I27.82, I27.83, I27.89
